# Crystal structure of sunvozertinib, C_29_H_35_ClFN_7_O_3_, from synchrotron X-ray powder data and DFT optimization

**DOI:** 10.1107/S2056989026004706

**Published:** 2026-05-15

**Authors:** Jacob K. Salazar, James A. Kaduk, Anja Dosen, Thomas N. Blanton

**Affiliations:** a North Central College, Department of Chemistry 131 S Loomis St Naperville IL 60540 USA; b Department of Physics North Central College, 131 S Loomis St Naperville IL 60540 USA; c ICDD, 12 Campus Blvd., Newtown Square, PA 19073, USA; University of Aberdeen, United Kingdom

**Keywords:** powder diffraction, sunvozertinib, Zegfrovy, Rietveld refinement, density functional theory

## Abstract

The crystal structure of sunvozertinib (systematic name: *N*-[5-({4-[5-chloro-4-fluoro-2-(2-hydroxypropan-2-yl)anilino]pyrimidin-2-yl}amino)-2-[(3*R*)-3-(dimethylamino)pyrrolidin-1-yl]-4-methoxyphenyl]prop-2-enamide), C_29_H_35_ClFN_7_O_3_, has been solved and refined using synchrotron X-ray powder diffraction data, and optimized using density functional theory techniques. The asymmetric unit in space group *C*2 contains two mol­ecules, *A* and *B*, and the crystal structure consists of alternating layers of mol­ecules *A* and *B* lying parallel to (201). O—H⋯O hydrogen bonds link the *B* mol­ecules into chains propagating along the *b*-axis direction while pairwise N—H⋯N hydrogen bonds link the *A* mol­ecules into dimers.

## Abstract 2

The crystal structure of sunvozertinib has been solved and refined using synchrotron X-ray powder diffraction data, and optimized using density functional theory techniques.

## 1. Chemical context

Sunvozertinib (C_29_H_35_ClFN_7_O_3_; marketed as Zegfrovy) is used to treat non-small-cell lung cancer (Wang *et al.*, 2022 ▸). It is administered to adult patients with locally advanced or metastatic non-small-cell lung cancer (NSCLC) when the disease has progressed on or after platinum-based chemotherapy. Its systematic name (CAS Registry Number 2370013-12-8) is *N*-[5-[[4-[5-chloro-4-fluoro-2-(2-hy­droxy­propan-2-yl)anilino]pyrimidin-2-yl]amino]-2-[(3*R*)-3-(di­methyl­amino)­pyr­rolidin-1-yl]-4-meth­oxy­phen­yl]prop-2-enamide.
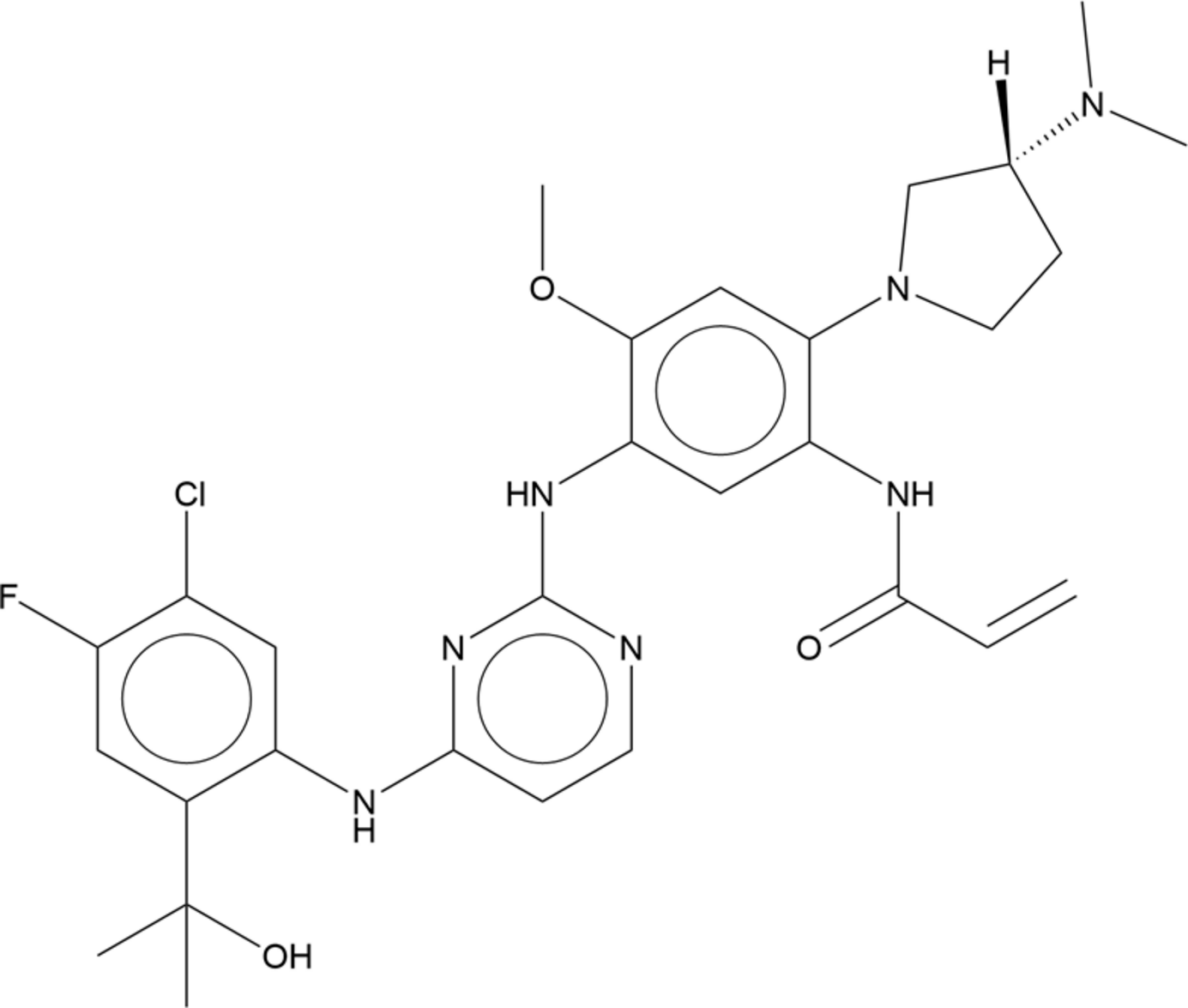


This work was carried out as part of a project (Kaduk *et al.*, 2014 ▸) to determine the crystal structures of large-volume commercial pharmaceuticals, and include high-quality powder diffraction data for them in the Powder Diffraction File (Kabekkodu *et al.*, 2024 ▸).

## 2. Structural commentary

**Figure 1:**
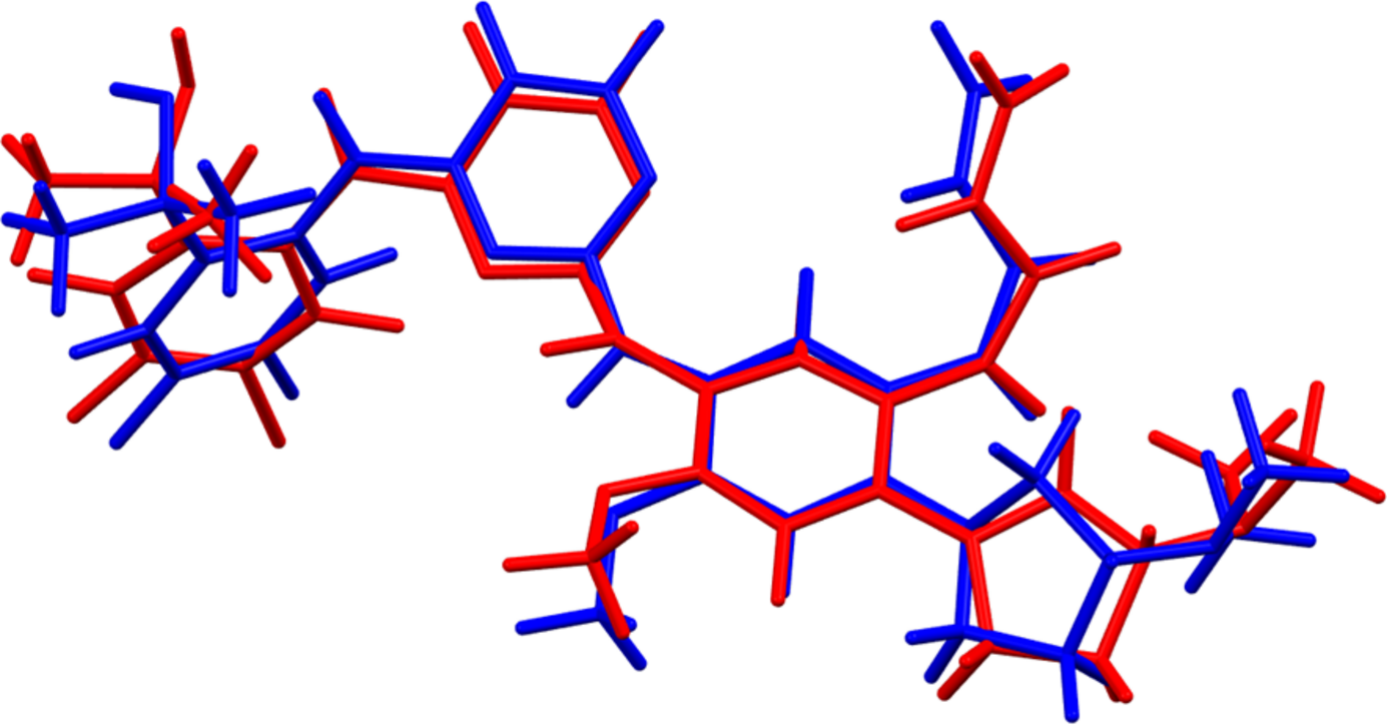
Comparison of the refined structure of sunvozertinib mol­ecule *A* (red) to the *VASP*-optimized structure (blue). The comparison was generated using the *Mercury* Calculate/Mol­ecule overlay tool; the r.m.s. difference is 0.838 Å.

**Figure 3:**
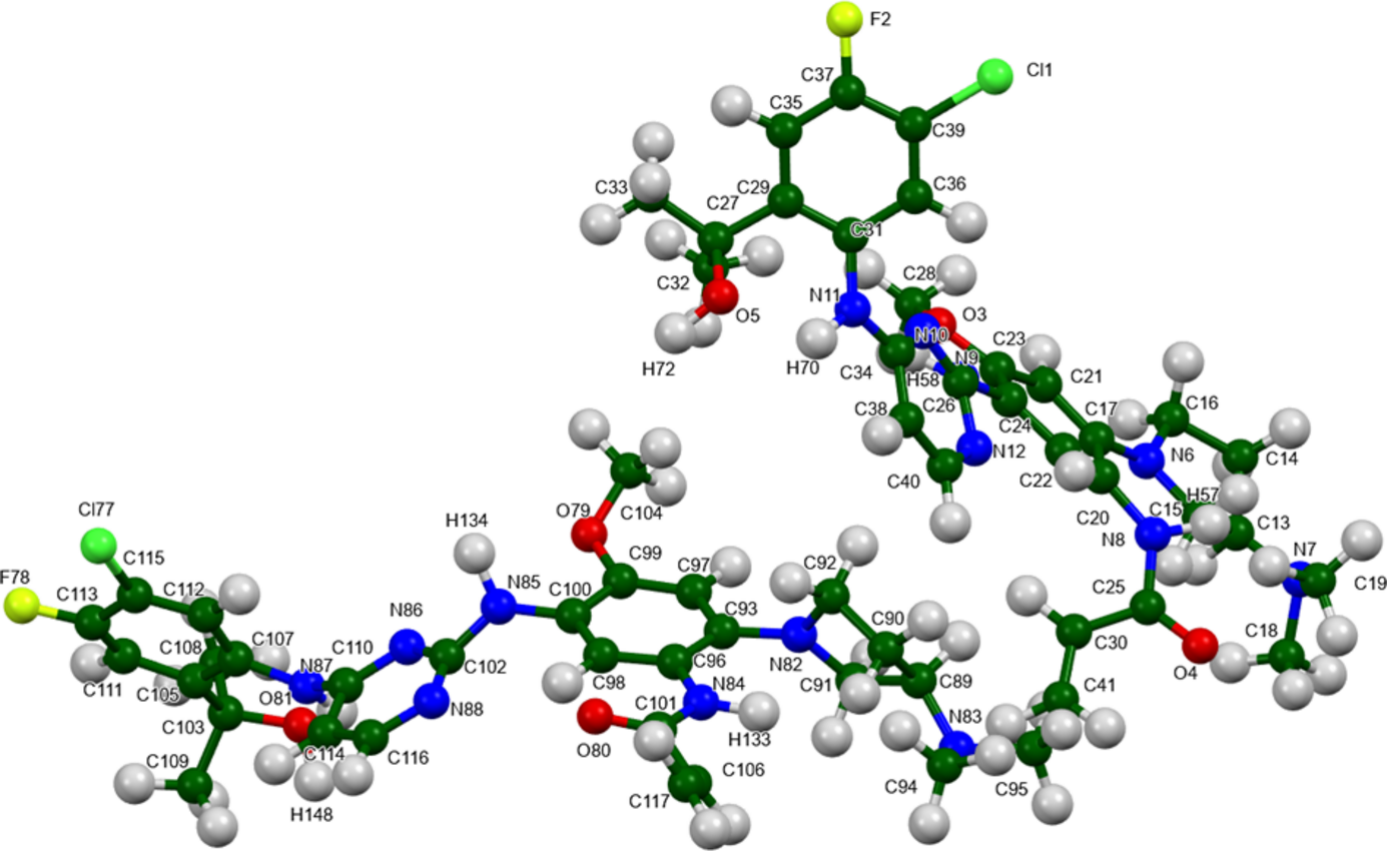
The asymmetric unit of sunvozertinib, with the atom numbering. The atoms are represented by 50% probability spheroids.

Sunvozertinib crystallises in the monoclinic space group *C*2 with two mol­ecules, *A* and *B*, in the asymmetric unit. The root-mean-square Cartesian displacements of the non-H atoms in the Rietveld-refined and DFT-optimized structures of mol­ecules *A* and *B*, calculated using the *Mercury* Calculate/Mol­ecule overlay tool (Macrae *et al.*, 2020 ▸), are 0.838 and 0.636 Å, respectively (Figs. 1 ▸ and 2 ▸). The differences are spread throughout the mol­ecules. The agreements are outside of the normal range for correct structures (van de Streek & Neumann, 2014 ▸); however, this very complex structure, refined using limited data, might be expected to be less accurate than usual. In the refined structure, there is a close contact (overlap) between the vinyl group C95 of mol­ecule *A* and one of the methyl­amine groups associated with atom N83 of mol­ecule *B*. This contact is relieved on DFT optimization. The asymmetric unit is illustrated in Fig. 3 ▸. The remaining discussion will emphasize the *VASP*-optimized structure.

All of the bond distances, and most of the bond angles and torsion angles fall within the normal ranges indicated by a *Mercury* Mogul Geometry check (Macrae *et al.*, 2020 ▸). The angles C3—C25—N8 [116.2°; average = 113.8 (7)°; *Z*-score = 3.4], C104—O79—C99 [112.2°; average = 117.5 (15)°; *Z*-score = 3.5], and O79—C99—C100 [119.3°; average = 114.8 (12)°; *Z*-score = 3.7] are flagged as unusual. For all three, the uncertainty on average is exceptionally small, inflating the *Z*-scores, so these are not of concern. Torsion angles involving rotation about the C13—N7 and C89—N83 bonds (which reflect the orientations of the di­methyl­amino groups in the two mol­ecules) lie in minor *trans* populations of a mainly *gauche* distribution. The torsion angles about C93—N82 lie in the middle of broad ranges. Torsion angles involving C110—N87 lie on the tails of distributions, so they are slightly unusual. Torsions about C25—N8 (amide) and O79—C99 (meth­oxy) are flagged as unusual.

**Figure 4:**
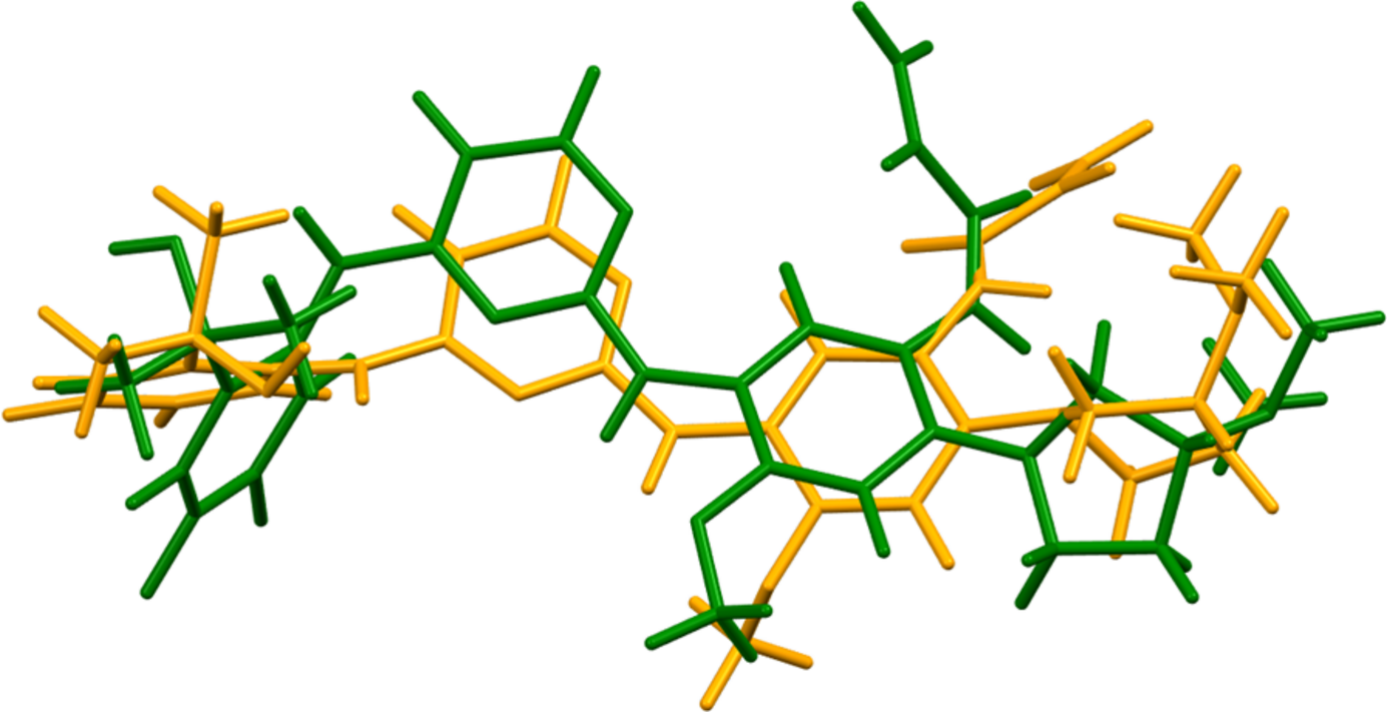
Comparison of the *VASP*-optimized structures of sunvozertinib mol­ecule *A* (green) and mol­ecule *B* (orange). The r.m.s. difference is 1.706 Å.

The root-mean-square difference between mol­ecules *A* and *B* is 1.706 Å (Fig. 4 ▸). As noted above, the differences are spread throughout the mol­ecules. The inter­planar angles between the aromatic rings in mol­ecule *A* are 64.8 and 27.2°, and those in mol­ecule *B* are 66.7 and 31.3°. Quantum chemical geometry optimization of the isolated sunvozertinib mol­ecules (DFT/B3LYP/6-31G*/water) using *Spartan ’24* (Wavefunction, 2025 ▸) indicated that mol­ecule *B* is 1.8 kcal mol^−1^ lower in energy than mol­ecule *A*. Since the expected uncertainty of such calculations is of the order of 1 kcal mol^−1^, the two mol­ecules should be considered to be equivalent in energy. The mol­ecule is apparently flexible: the global minimum-energy conformation is 233 kcal mol^−1^ lower in energy, but is much more compact, being folded on itself. Inter­molecular inter­actions are thus important in determining the solid-state conformation.

## 3. Supra­molecular features

**Figure 5:**
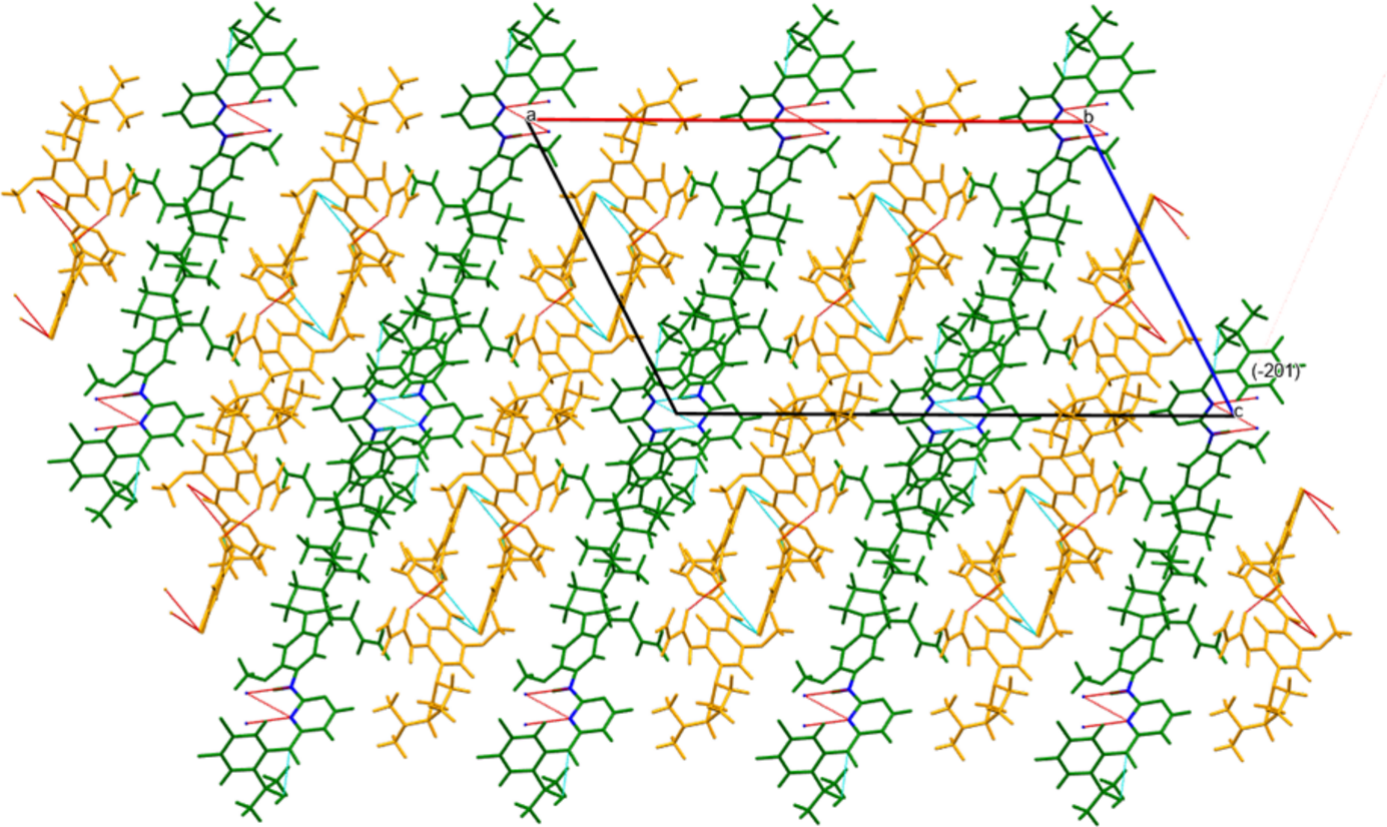
Crystal structure of sunvozertinib, viewed down the *b*-axis direction. Mol­ecule *A* is green, and mol­ecule *B* is orange.

The extended structure (Fig. 5 ▸) consists of alternating layers of mol­ecules *A* and *B* lying parallel to the (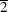
01) plane. O—H⋯O hydrogen bonds (Table 1 ▸) link the *B* mol­ecules into chains propagating along the *b*-axis direction. N—H⋯N hydrogen bonds link the *A* mol­ecules into pairs. The *Mercury* Aromatics Analyser indicates two strong (*d* = 4.96 Å) inter­actions between the *A* mol­ecules, and two moderate (*d* = 4.96 Å) parallel stacking inter­actions between the *B* mol­ecules.

Analysis of the contributions to the total crystal energy of the structure using the Forcite module of *Materials Studio* (Dassault Systèmes, 2024 ▸) indicated that the intra­molecular energy is dominated by torsion angle distortion terms, with a significant contribution from angle distortion terms. The inter­molecular energy is dominated by van der Waals attractions, which in this force-field-based analysis include hydrogen bonds. The hydrogen bonds are better discussed using the results of the DFT calculation.

Hydrogen bonds are prominent in the structure. Strong O81—H148⋯O80 hydrogen bonds link the *B* mol­ecules into chains along the *b*-axis direction. The graph set descriptor (Etter, 1990 ▸; Bernstein *et al.*, 1995 ▸; Motherwell *et al.*, 2000 ▸) for this pattern is 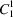
(16). The energy of the O—H⋯O hydrogen bond (O81—H148⋯O80 = 13.2 kcal mol^−1^) was calculated using the correlation of Rammohan and Kaduk (2018 ▸). There are two intra­molecular N—H⋯O hydrogen bonds in mol­ecule *B*. The energy of the N87—H146⋯O81 hydrogen bond (5.4 kcal mol^−1^) was calculated using the correlation of Wheatley and Kaduk (2019 ▸).

Pairwise N9—H58⋯N10 bonds link the *A* mol­ecules into dimers with crystallographic twofold symmetry, with a graph-set notation of 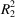
(8). The O5—H72 group of mol­ecule *A* does not form a hydrogen bond in the present model although an alternative orientation that would form an inter­molecular O5—H72⋯Cl77 link is possible.

Intra­molecular N—H⋯N bonds are present in both mol­ecules. N—H⋯Cl bonds also participate in the chains of mol­ecules *B*. Several weak C—H⋯N and C—H⋯O hydrogen bonds also contribute to the cohesion of the structure.

**Figure 6:**
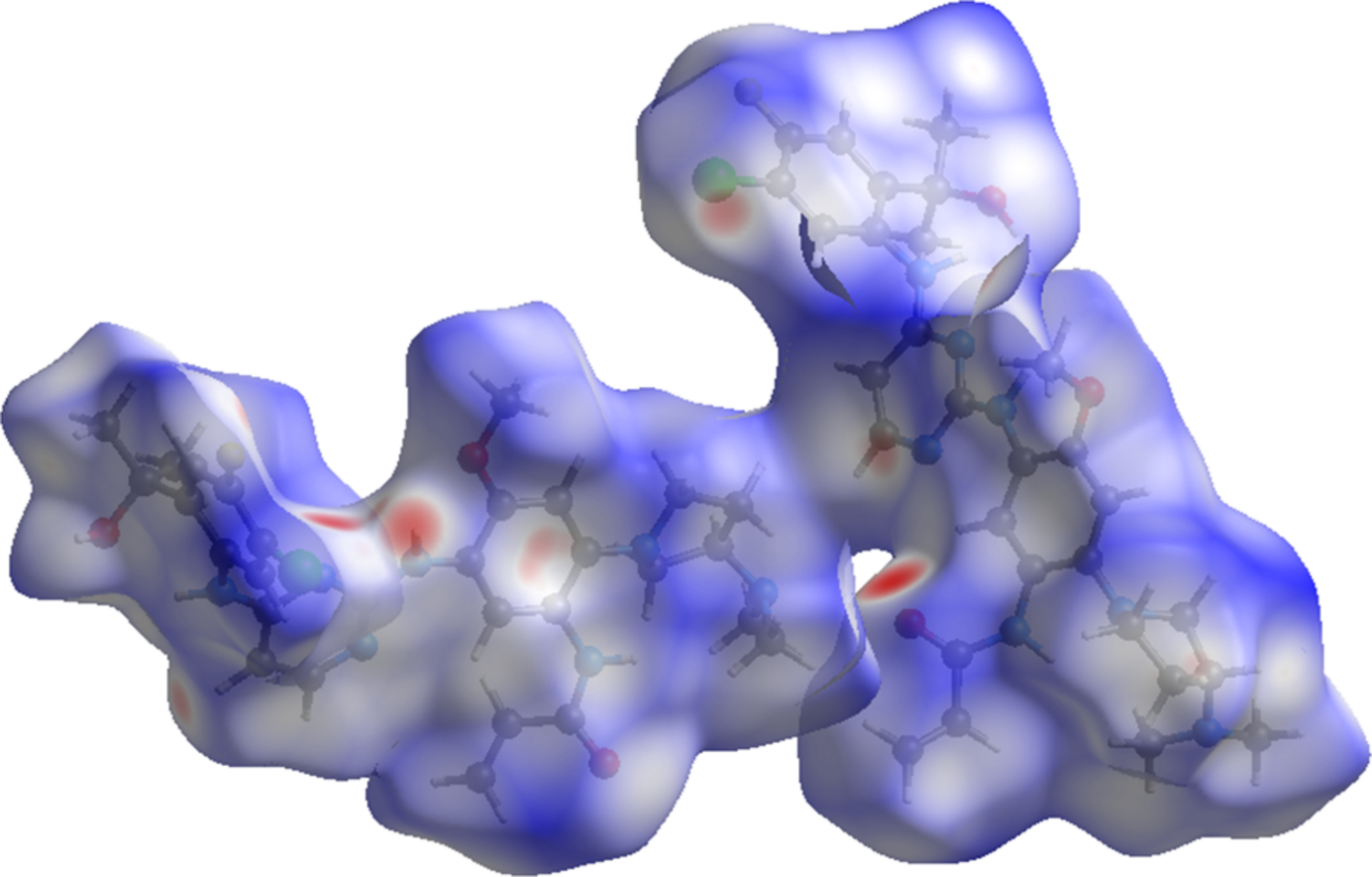
The Hirshfeld surface of sunvozertinib. Inter­molecular contacts longer than the sums of the van der Waals radii are colored blue, and contacts shorter than the sums of the radii are colored red. Contacts equal to the sums of radii are white.

The volume enclosed by the Hirshfeld surface of sunvozertinib (Fig. 6 ▸; Hirshfeld, 1977 ▸; Spackman *et al.*, 2021 ▸) is 1491.6 Å^3^, some 98.7% of 1/4 of the unit-cell volume. The packing density is thus typical. The only significant close contacts (red in Fig. 6 ▸) involve the hydrogen bonds. The volume per non-hydrogen atom is normal, at 18.4 Å^3^.

The Bravais–Friedel–Donnay–Harker (Bravais, 1866 ▸; Friedel, 1907 ▸; Donnay & Harker, 1937 ▸) algorithm suggests that we might expect elongated morphology for sunvozertinib, with [010] as the long axis. A 2nd order spherical harmonic model for preferred orientation was included. The texture index was 1.036 (3), indicating that the preferred orientation was small in this rotated capillary specimen.

## 4. Database survey

A reduced cell search in the Cambridge Structural Database (CSD Conquest Build 2026.1.0; Groom *et al.*, 2016 ▸) yielded one hit for an unrelated structure, but no structures of sunvozertinib or its derivatives. We are unaware of any published X-ray powder diffraction data for sunvozertinib.

## 5. Synthesis and crystallization

Sunvozertinib is a commercial reagent, purchased from TargetMol (Batch #231941), and was used as-received.

## 6. Refinement

Crystal data, data collection and structure refinement details are summarized in Table 2 ▸. The white powder was packed into a 0.5 mm diameter Kapton capillary, and rotated during the measurements at ∼2 Hz. The powder pattern was measured at 298 (1) K at the Wiggler Low Energy Beamline (Leontowich *et al.*, 2021 ▸) of the Brockhouse X-ray Diffraction and Scattering Sector of the Canadian Light Source using a wavelength of 0.819325 (2) Å (15.1 keV) from 1.6–75.0° 2θ with a step size of 0.0025° and a collection time per step of 3 minutes. The high-resolution powder diffraction data were collected using eight Dectris Mythen2 X series 1K linear strip detectors. NIST SRM 660b LaB_6_ was used to calibrate the instrument and refine the monochromatic wavelength used in the experiment.

The pattern was indexed using *JADE Pro* (MDI, 2025 ▸) on a *C*-centered monoclinic cell with *a* = 33.43691, *b* = 10.20685, *c* = 19.80699 Å, *β* = 117.27°, *V* = 6008.62 Å^3^, and *Z* = 8. The space group suggested by *EXPO2014* (Altomare *et al.*, 2013 ▸) was *C2*, which was confirmed by the successful solution and refinement of the structure.

The mol­ecular structure of sunvozertinib was downloaded from PubChem (Kim *et al.*, 2023 ▸) as Conformer3D_COMPOUND_CID_139377809.sdf. It was converted to a *.mol2 file using *Mercury* (Macrae *et al.*, 2020 ▸). The crystal structure was solved by Monte Carlo simulated annealing techniques as implemented in *EXPO2014* (Altomare *et al.*, 2013 ▸) using the two sunvozertinib mol­ecules as fragments, including a bump penalty on the non-H atoms.

Rietveld refinement was carried out with *GSAS-II* (Toby & Von Dreele, 2013 ▸). Only the 2.5–40.0° portion of the pattern was included in the refinements (*d_min_* = 1.198 Å). All non-H bond distances and angles were subjected to restraints, based on a *Mercury* Mogul Geometry Check (Sykes *et al.*, 2011 ▸; Bruno *et al.*, 2004 ▸). The Mogul average and standard deviation for each qu­antity were used as the restraint parameters. The aromatic rings were restrained to be planar. The restraints contributed 13.6% to the overall *χ^2^*. Decreasing the restraint weights led to disconnected mol­ecular fragments. The hydrogen atoms were included in calculated positions, which were recalculated during the refinement using *Materials Studio* (Dassault Systèmes, 2024 ▸). Attempts to refine isotropic displacement coefficients (grouped by chemical similarity) led to unreasonably-large positive and negative values, so the *U*_iso_ were fixed at reasonable values. The peak profiles were described using a uniaxial microstrain model, with [010] as the unique axis. The background was modeled using a six-term shifted Chebyshev polynomial, with two peaks at 3.05 and 10.87° 2θ to model the scattering from the Kapton capillary and any amorphous component of the sample.

**Figure 7:**
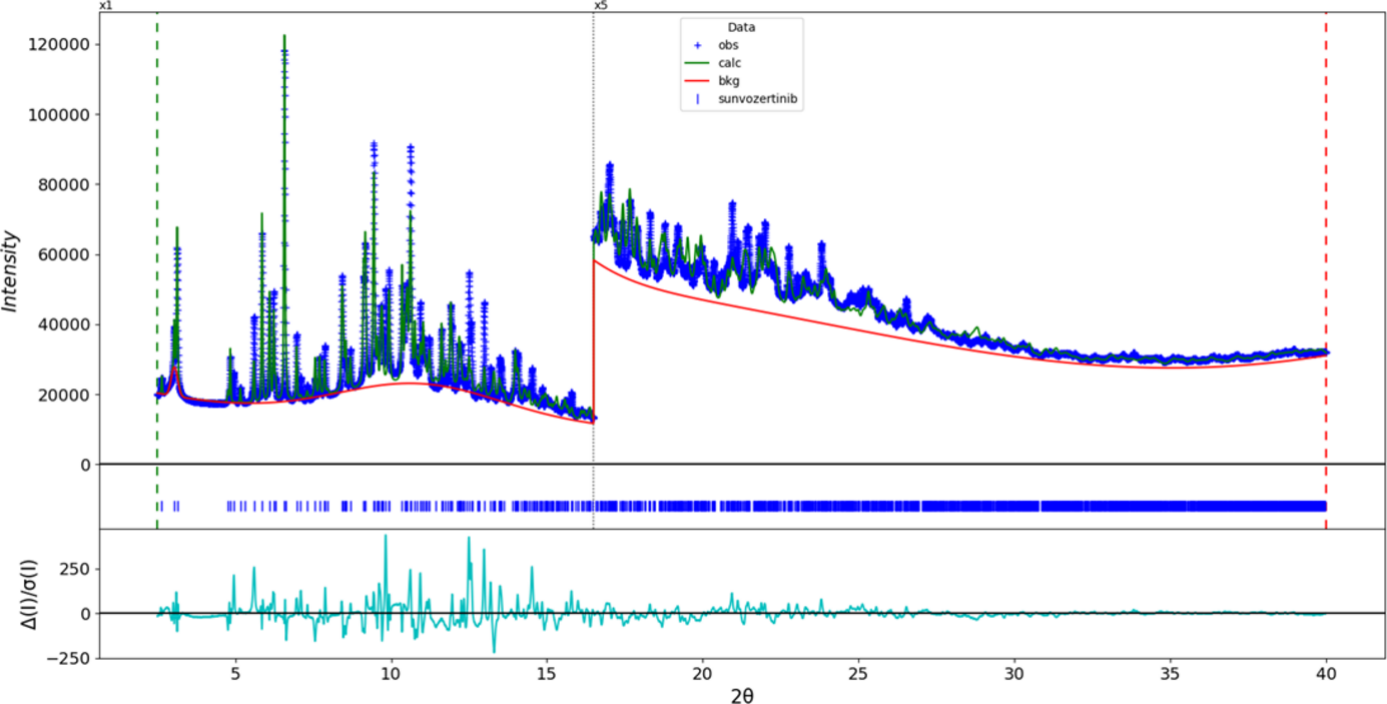
The Rietveld difference plot for sunvozertinib. The blue crosses represent the observed data points, and the green line is the calculated pattern. The cyan curve is the normalized error plot, and the red line is the background curve. The blue tick marks indicate the peak positions. The vertical scale has been multiplied by a factor of 5 for 2θ > 16.5°.

The final refinement of 268 variables using 15,001 observations and 222 restraints yielded the residual *R*_wp_ = 0.0993. The largest peak (1.42 Å from C15) and hole (2.15 Å from C93) in the difference-Fourier map are +0.53 (13) and −0.47 (13) e Å^−3^, respectively. The final Rietveld plot is shown in Fig. 7 ▸. The largest features in the normalized error plot are in the intensities and shapes of some of the strong low-angle peaks.

The crystal structure of sunvozertinib was optimized (fixed experimental unit cell) with density functional theory techniques using *VASP* (Kresse and Furthmüller, 1996 ▸) through the *MedeA* graphical inter­face (Materials Design, 2024 ▸). The calculation was carried out on 32 cores of a 144-core (768 Gb memory) HPE Superdome Flex 280 Linux server at North Central College. The calculation used the GGA-PBE functional, a plane wave cutoff energy of 400.0 eV, and a *k*-point spacing of 0.5 Å^−1^ leading to a 3 × 3 × 1 mesh, and took ∼2.9 days. Single-point density functional theory calculations (fixed experimental cell) and population analysis were carried out using *CRYSTAL23* (Erba *et al.*, 2023 ▸). The basis sets for the H, C, N and O atoms in the calculation were those of Gatti *et al.* (1994 ▸), and those for F and Cl were from Peintinger *et al.* (2013 ▸). The calculations were run on a 3.5 GHz PC using 8 *k*-points and the B3LYP functional, and took ∼11.4 h.

## Supplementary Material

Crystal structure: contains datablock(s) I, _I_VASP. DOI: 10.1107/S2056989026004706/hb8203sup1.cif

CCDC references: 2552040, 2552041

Additional supporting information:  crystallographic information; 3D view; checkCIF report

## Figures and Tables

**Figure 2:**
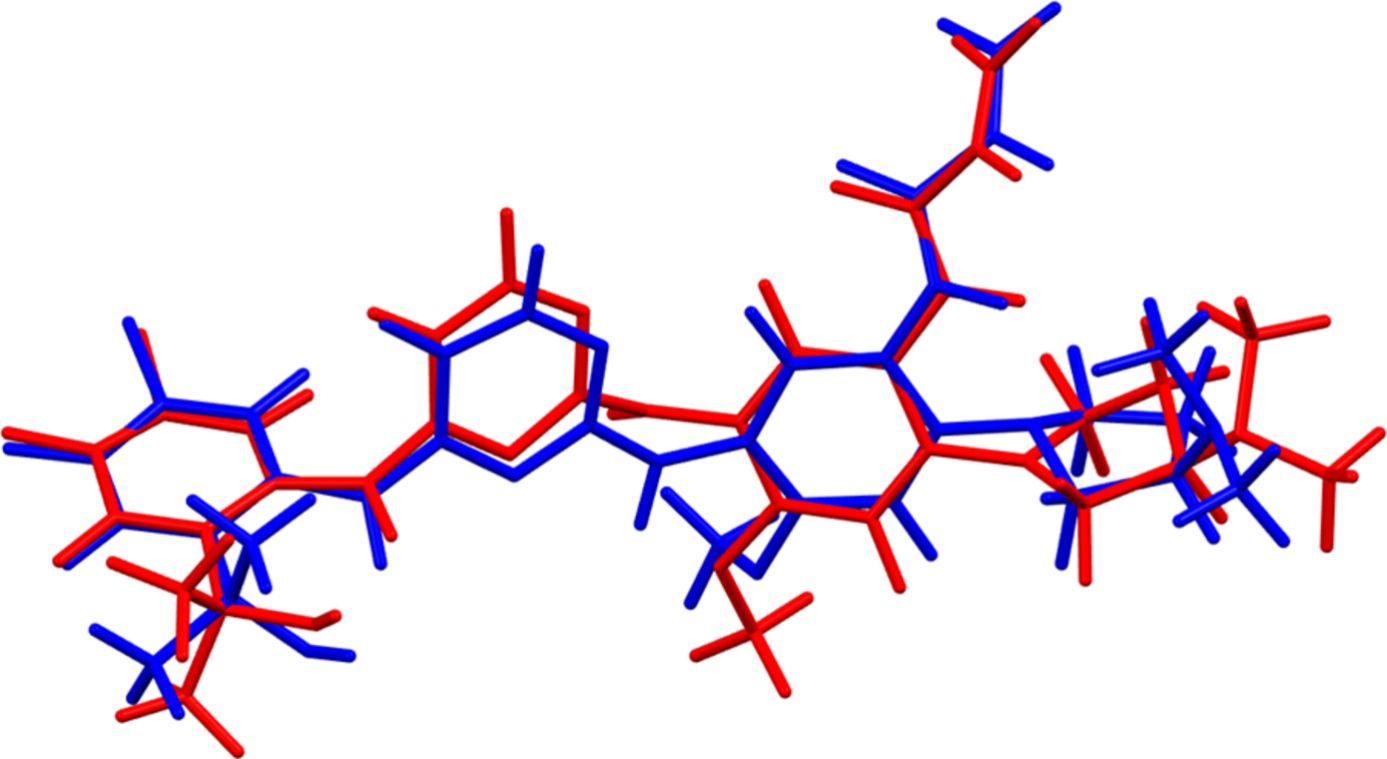
Comparison of the refined structure of sunvozertinib mol­ecule *B* (red) to the *VASP*-optimized structure (blue). The comparison was generated using the *Mercury* Calculate/Mol­ecule overlay tool; the r.m.s. difference is 0.636 Å.

**Table 1:** Hydrogen-bond geometry (Å, °)

*D*—H⋯*A*	*D*—H	H⋯*A*	*D*⋯*A*	*D*—H⋯*A*
N9—H58⋯N10^i^	1.03	2.10	3.022	148
N11—H70⋯O5	1.02	2.18	2.872	124
N11—H70⋯Cl77^ii^	1.02	2.82	3.597	133
N85—H134⋯Cl77^iii^	1.02	2.47	3.424	155
N87—H146⋯O81	1.03	1.96	2.705	127
O81—H148⋯O80^iv^	0.99	1.77	2.740	164
C38—H73⋯O79^v^	1.09	2.32	3.329	154
C90—H120⋯N12^vi^	1.10	2.51	3.358	133
C104—H136⋯O5^vii^	1.10	2.53	3.498	147
C108—H141⋯N88^iv^	1.10	2.33	3.400	165

Symmetry codes: (i) 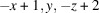; (ii) 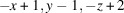; (iii) 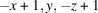; (iv) 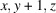; (v) 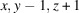; (vi) 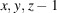; (vii) 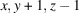.

**Table 2:** Experimental details

Crystal data	
Chemical formula	C_29_H_35_ClFN_7_O_3_
*M*_r_	584.09
Crystal system, space group	Monoclinic, *C*2
Temperature (K)	298
*a*, *b*, *c* (Å)	33.491 (9), 10.2237 (6), 19.857 (4)
β (°)	117.216 (9)
*V* (Å^3^)	6046.4 (10)
*Z*	8
Radiation type	Synchrotron, λ = 0.81933 Å
μ (mm^−1^)	0.11
Specimen shape, size (mm)	Cylinder, 0.45 × 0.15

Data collection	
Diffractometer	Wiggler Low Energy Beamline, Brockhouse X-ray Diffraction and Scattering Sector, Canadian Light Source
Specimen mounting	Kapton capillary
Data collection mode	Transmission
Scan method	Step
2θ values (°)	2θ_min_ = 1.6, 2θ_max_ = 75.0, 2θ_step_ = 0.003

Refinement	
*R* factors and goodness of fit	*R*_p_ = 0.061, *R*_wp_ = 0.0993, *R*_exp_ = 0.002, *R*(*F*^2^) = 0.26899, χ^2^ = 2361.571
No. of parameters	268
No. of restraints	222
(Δ/σ)_max_	4.249

Computer programs: *GSAS-II* (Toby & Von Dreele, 2013 ▸).

## supplementary crystallographic information

### N-[5-({4-[5-Chloro-4-fluoro-2-(2-hydroxypropan-2-yl)anilino]pyrimidin-2-yl}amino)-2-[(3R)-3-(dimethylamino)pyrrolidin-1-yl]-4-methoxyphenyl]prop-2-enamide (I) Crystal data

**Table:**

C_29_H_35_ClFN_7_O_3_	*V* = 6046.4 (10) Å^3^
*M**_r_* = 584.09	*Z* = 8
Monoclinic, *C*2	*D*_x_ = 1.283 Mg m^−^^3^
*a* = 33.491 (9) Å	Synchrotron radiation, λ = 0.81933 Å
*b* = 10.2237 (6) Å	µ = 0.11 mm^−^^1^
*c* = 19.857 (4) Å	*T* = 298 K
β = 117.216 (9)°	cylinder, 0.45 × 0.15 mm

### N-[5-({4-[5-Chloro-4-fluoro-2-(2-hydroxypropan-2-yl)anilino]pyrimidin-2-yl}amino)-2-[(3R)-3-(dimethylamino)pyrrolidin-1-yl]-4-methoxyphenyl]prop-2-enamide (I) Data collection

**Table:**

Wiggler Low Energy Beamline, Brockhouse X-ray Diffraction and Scattering Sector, Canadian Light Source diffractometer	Scan method: step
Specimen mounting: Kapton capillary	2θ_min_ = −9.008°, 2θ_max_ = 75.047°, 2θ_step_ = 0.003°
Data collection mode: transmission	

### N-[5-({4-[5-Chloro-4-fluoro-2-(2-hydroxypropan-2-yl)anilino]pyrimidin-2-yl}amino)-2-[(3R)-3-(dimethylamino)pyrrolidin-1-yl]-4-methoxyphenyl]prop-2-enamide (I) Refinement

**Table:**

Least-squares matrix: full	268 parameters
*R*_p_ = 0.061	222 restraints
*R*_wp_ = 0.092	0 constraints
*R*_exp_ = 0.002	Weighting scheme based on measured s.u.'s
*R*(*F*^2^) = 0.26899	(Δ/σ)_max_ = 4.249
33623 data points	Background function: Background function: "chebyschev-1" function with 6 terms: 1.038(5)e4, -6.46(6)e3, 2.14(4)e3, -3.6(8)e2, 6.9(7)e2, -1.9(3)e2, Background peak parameters: pos, int, sig, gam: 3.050(5), 2.13(10)e5, 103(9), 0.100, 10.894(31), 7.23(23)e6, 7.23(23)e4, 0.100,
Profile function: Finger-Cox-Jephcoat function parameters U, V, W, X, Y, SH/L: peak variance(Gauss) = Utan(Th)^2^+Vtan(Th)+W: peak HW(Lorentz) = X/cos(Th)+Ytan(Th); SH/L = S/L+H/L U, V, W in (centideg)^2^, X & Y in centideg 6.157, -1.198, 1.258, 0.000, 0.667, 0.002,	Preferred orientation correction: Simple spherical harmonic correction Order = 2 Coefficients: 0:0:C(2,-2) = 0.126(18); 0:0:C(2,0) = -0.206(24); 0:0:C(2,2) = 0.270(18)

### N-[5-({4-[5-Chloro-4-fluoro-2-(2-hydroxypropan-2-yl)anilino]pyrimidin-2-yl}amino)-2-[(3R)-3-(dimethylamino)pyrrolidin-1-yl]-4-methoxyphenyl]prop-2-enamide (I) Fractional atomic coordinates and isotropic or equivalent isotropic displacement parameters (Å^2^)

**Table:**

	*x*	*y*	*z*	*U*_iso_*/*U*_eq_	
Cl1	0.6105 (9)	−0.58413	1.0397 (13)	0.0500*	
F2	0.6809 (9)	−0.518 (6)	1.1997 (16)	0.0500*	
O3	0.4737 (10)	0.158 (4)	0.9250 (19)	0.0500*	
O4	0.2231 (10)	−0.037 (5)	0.6534 (18)	0.0500*	
O5	0.5494 (17)	−0.365 (6)	1.294 (3)	0.0500*	
N6	0.3421 (9)	0.219 (4)	0.6663 (15)	0.0500*	
N7	0.2348 (11)	0.211 (5)	0.4865 (16)	0.0500*	
N8	0.2994 (9)	0.021 (4)	0.7000 (14)	0.0500*	
N9	0.4386 (15)	−0.065 (4)	0.9464 (16)	0.0500*	
N10	0.4777 (8)	−0.241 (4)	1.0326 (19)	0.0500*	
N11	0.5090 (9)	−0.377 (5)	1.1443 (15)	0.0500*	
N12	0.3964 (8)	−0.225 (6)	0.964 (3)	0.0500*	
C13	0.2774 (9)	0.263 (4)	0.5502 (16)	0.0500*	
C14	0.3201 (12)	0.301 (7)	0.5406 (18)	0.0500*	
C15	0.2969 (11)	0.164 (5)	0.6150 (18)	0.0500*	
C16	0.3597 (11)	0.298 (5)	0.622 (2)	0.0500*	
C17	0.3678 (11)	0.154 (5)	0.739 (2)	0.0500*	
C18	0.1927 (12)	0.269 (7)	0.485 (4)	0.0500*	
C19	0.2307 (19)	0.078 (5)	0.496 (4)	0.0500*	
C20	0.3459 (8)	0.050 (4)	0.7550 (17)	0.0500*	
C21	0.4122 (13)	0.191 (5)	0.794 (2)	0.0500*	
C22	0.3712 (13)	−0.029 (4)	0.818 (2)	0.0500*	
C23	0.4335 (17)	0.122 (5)	0.863 (2)	0.0500*	
C24	0.4139 (14)	0.006 (5)	0.874 (2)	0.0500*	
C25	0.2585 (8)	0.013 (4)	0.7043 (16)	0.0500*	
C26	0.4381 (9)	−0.192 (4)	0.9756 (18)	0.0500*	
C27	0.5832 (10)	−0.292 (3)	1.2855 (16)	0.0500*	
C28	0.491 (2)	0.286 (5)	0.927 (3)	0.0500*	
C29	0.5891 (8)	−0.370 (5)	1.2248 (17)	0.0500*	
C30	0.2623 (15)	0.041 (9)	0.781 (3)	0.0500*	
C31	0.5527 (8)	−0.391 (5)	1.1534 (15)	0.0500*	
C32	0.567 (3)	−0.153 (4)	1.258 (3)	0.0500*	
C33	0.6256 (12)	−0.289 (7)	1.361 (2)	0.0500*	
C34	0.4728 (8)	−0.327 (7)	1.080 (3)	0.0500*	
C35	0.6305 (9)	−0.427 (5)	1.2429 (13)	0.0500*	
C36	0.5599 (9)	−0.445 (7)	1.0942 (15)	0.0500*	
C37	0.6374 (9)	−0.484 (9)	1.1849 (18)	0.0500*	
C38	0.4311 (10)	−0.361 (9)	1.074 (4)	0.0500*	
C39	0.6023 (8)	−0.498 (5)	1.1111 (13)	0.0500*	
C40	0.3940 (8)	−0.305 (9)	1.015 (4)	0.0500*	
C41	0.227 (2)	0.016 (10)	0.798 (3)	0.0500*	
H42	0.26773	0.35323	0.57321	0.0650*	
H43	0.32498	0.22679	0.50215	0.0650*	
H44	0.31608	0.40347	0.52569	0.0650*	
H45	0.30100	0.06274	0.59493	0.0650*	
H46	0.27521	0.15850	0.64551	0.0650*	
H47	0.39018	0.24802	0.62253	0.0650*	
H48	0.36898	0.40040	0.64795	0.0650*	
H49	0.17173	0.31755	0.42782	0.0650*	
H50	0.17226	0.18806	0.49413	0.0650*	
H51	0.20247	0.34496	0.53193	0.0650*	
H52	0.24345	0.02044	0.46055	0.0650*	
H53	0.19392	0.05303	0.47699	0.0650*	
H54	0.25135	0.05129	0.55827	0.0650*	
H55	0.43002	0.27487	0.78049	0.0650*	
H56	0.35699	−0.12617	0.82585	0.0650*	
H57	0.29101	−0.00079	0.64024	0.0650*	
H58	0.46590	−0.01532	0.99563	0.0650*	
H59	0.46572	0.36177	0.92554	0.0650*	
H60	0.49718	0.30010	0.87529	0.0650*	
H61	0.52393	0.29850	0.98065	0.0650*	
H62	0.29468	0.08373	0.82705	0.0650*	
H63	0.56038	−0.14227	1.19667	0.0650*	
H64	0.59364	−0.07973	1.29487	0.0650*	
H65	0.53452	−0.13347	1.26195	0.0650*	
H66	0.62020	−0.22161	1.40195	0.0650*	
H67	0.63346	−0.39198	1.38574	0.0650*	
H68	0.65485	−0.25122	1.35216	0.0650*	
H69	0.65805	−0.42674	1.30400	0.0650*	
H70	0.50097	−0.40691	1.19047	0.0650*	
H71	0.53158	−0.44526	1.03372	0.0650*	
H72	0.53887	−0.31169	1.33145	0.0650*	
H73	0.42873	−0.43182	1.11637	0.0650*	
H74	0.35995	−0.33061	1.01142	0.0650*	
H75	0.19925	−0.05697	0.76289	0.0650*	
H76	0.22510	0.06878	0.84758	0.0650*	
Cl77	0.5504 (9)	0.766 (3)	0.7381 (12)	0.0500*	
F78	0.5324 (12)	1.048 (3)	0.7594 (18)	0.0500*	
O79	0.4365 (16)	0.507 (5)	0.236 (2)	0.0500*	
O80	0.3326 (17)	0.085 (6)	0.3147 (16)	0.0500*	
O81	0.3926 (16)	1.086 (4)	0.4255 (16)	0.0500*	
N82	0.3283 (11)	0.178 (4)	0.0645 (13)	0.0500*	
N83	0.2235 (11)	0.038 (4)	−0.090 (2)	0.0500*	
N84	0.314 (2)	0.098 (6)	0.1912 (16)	0.0500*	
N85	0.4176 (10)	0.443 (4)	0.3499 (17)	0.0500*	
N86	0.415 (2)	0.649 (3)	0.406 (3)	0.0500*	
N87	0.4160 (12)	0.842 (3)	0.4758 (15)	0.0500*	
N88	0.3794 (19)	0.452 (4)	0.420 (3)	0.0500*	
C89	0.2725 (11)	0.076 (4)	−0.0516 (16)	0.0500*	
C90	0.2998 (14)	−0.026 (4)	0.011 (3)	0.0500*	
C91	0.2877 (14)	0.211 (3)	−0.007 (2)	0.0500*	
C92	0.3436 (10)	0.047 (4)	0.059 (3)	0.0500*	
C93	0.3489 (14)	0.250 (5)	0.1366 (13)	0.0500*	
C94	0.2177 (18)	−0.078 (7)	−0.051 (4)	0.0500*	
C95	0.2047 (19)	0.005 (8)	−0.171 (2)	0.0500*	
C96	0.343 (2)	0.206 (6)	0.1991 (19)	0.0500*	
C97	0.3782 (19)	0.354 (6)	0.1475 (15)	0.0500*	
C98	0.365 (3)	0.273 (7)	0.268 (2)	0.0500*	
C99	0.4023 (12)	0.414 (5)	0.2184 (14)	0.0500*	
C100	0.3942 (18)	0.377 (5)	0.2791 (19)	0.0500*	
C101	0.3093 (13)	0.049 (5)	0.2491 (15)	0.0500*	
C102	0.4009 (14)	0.521 (3)	0.389 (2)	0.0500*	
C103	0.4180 (8)	1.143 (3)	0.4993 (16)	0.0500*	
C104	0.464 (3)	0.506 (8)	0.194 (5)	0.0500*	
C105	0.4454 (16)	1.039 (3)	0.5595 (16)	0.0500*	
C106	0.2702 (13)	−0.038 (6)	0.226 (3)	0.0500*	
C107	0.4492 (13)	0.906 (3)	0.5442 (18)	0.0500*	
C108	0.4481 (17)	1.245 (5)	0.489 (4)	0.0500*	
C109	0.3830 (16)	1.211 (6)	0.516 (3)	0.0500*	
C110	0.4079 (15)	0.707 (4)	0.462 (2)	0.0500*	
C111	0.4774 (13)	1.088 (3)	0.6320 (16)	0.0500*	
C112	0.483 (2)	0.828 (4)	0.5983 (18)	0.0500*	
C113	0.5053 (12)	1.002 (3)	0.6880 (15)	0.0500*	
C114	0.386 (3)	0.641 (5)	0.497 (4)	0.0500*	
C115	0.5117 (16)	0.876 (3)	0.6693 (15)	0.0500*	
C116	0.373 (3)	0.514 (6)	0.474 (4)	0.0500*	
C117	0.272 (2)	−0.144 (6)	0.259 (5)	0.0500*	
H118	0.28524	0.07349	−0.09588	0.0650*	
H119	0.30647	−0.11720	−0.01573	0.0650*	
H120	0.28062	−0.05220	0.04362	0.0650*	
H121	0.26006	0.25111	0.00512	0.0650*	
H122	0.29572	0.28388	−0.04306	0.0650*	
H123	0.36210	0.00034	0.11701	0.0650*	
H124	0.36636	0.05290	0.03009	0.0650*	
H125	0.21187	−0.16819	−0.08809	0.0650*	
H126	0.18756	−0.06257	−0.03974	0.0650*	
H127	0.24919	−0.09262	0.00504	0.0650*	
H128	0.20973	−0.10374	−0.17712	0.0650*	
H129	0.16729	0.02861	−0.20022	0.0650*	
H130	0.22245	0.06447	−0.19771	0.0650*	
H131	0.38313	0.39270	0.09798	0.0650*	
H132	0.35886	0.24272	0.31814	0.0650*	
H133	0.29429	0.05190	0.13494	0.0650*	
H134	0.45471	0.43582	0.38196	0.0650*	
H135	0.49728	0.55595	0.22973	0.0650*	
H136	0.46976	0.40065	0.18220	0.0650*	
H137	0.44497	0.56062	0.13813	0.0650*	
H138	0.23668	−0.00967	0.17655	0.0650*	
H139	0.56295	1.25964	0.57397	0.0650*	
H140	0.51558	1.20985	0.48189	0.0650*	
H141	0.55470	1.34162	0.48481	0.0650*	
H142	0.63000	1.30174	0.52090	0.0650*	
H143	0.64609	1.14119	0.49625	0.0650*	
H144	0.60104	1.24086	0.42162	0.0650*	
H145	0.47997	1.19761	0.64378	0.0650*	
H146	0.39318	0.90156	0.42637	0.0650*	
H147	0.48729	0.72246	0.58423	0.0650*	
H148	0.35653	1.10057	0.40394	0.0650*	
H149	0.37969	0.69231	0.54221	0.0650*	
H150	0.35576	0.46125	0.50417	0.0650*	
H151	0.30571	−0.17992	0.30551	0.0650*	
H152	0.24050	−0.20457	0.24252	0.0650*	

### N-[5-({4-[5-Chloro-4-fluoro-2-(2-hydroxypropan-2-yl)anilino]pyrimidin-2-yl}amino)-2-[(3R)-3-(dimethylamino)pyrrolidin-1-yl]-4-methoxyphenyl]prop-2-enamide (I) Geometric parameters (Å, º)

**Table:**

Cl1—C39	1.793 (11)	Cl77—C115	1.791 (11)
F2—C37	1.392 (17)	F78—C113	1.370 (18)
O3—C23	1.395 (8)	O79—C99	1.405 (8)
O3—C28	1.423 (14)	O79—C104	1.481 (14)
O4—C25	1.263 (10)	O80—C101	1.228 (10)
O5—C27	1.424 (7)	O81—C103	1.439 (8)
O5—H72	1.11 (5)	O81—H148	1.09 (5)
N6—C15	1.496 (9)	N82—C91	1.492 (9)
N6—C16	1.494 (8)	N82—C92	1.463 (8)
N6—C17	1.463 (11)	N82—C93	1.468 (11)
N7—C13	1.505 (10)	N83—H76^iii^	1.30 (4)
N7—C18	1.52 (2)	N83—C89	1.512 (10)
N7—C19	1.38 (2)	N83—C94	1.479 (18)
N8—C20	1.467 (10)	N83—C95	1.465 (18)
N8—C25	1.413 (8)	N84—C96	1.430 (9)
N8—H57	1.11 (3)	N84—C101	1.331 (8)
N9—C24	1.482 (13)	N84—H133	1.11 (3)
N9—C26	1.425 (11)	N85—C100	1.426 (13)
N9—H58	1.11 (3)	N85—C102	1.401 (11)
N10—C26	1.384 (11)	N85—H134	1.11 (3)
N10—C34	1.354 (13)	N86—C102	1.380 (11)
N11—C31	1.397 (13)	N86—C110	1.369 (9)
N11—C34	1.390 (9)	N87—C107	1.458 (13)
N11—H70	1.11 (3)	N87—C110	1.415 (9)
N12—C26	1.347 (6)	N87—H146	1.11 (3)
N12—C40	1.334 (10)	N88—C102	1.346 (6)
C13—N7	1.505 (10)	N88—C116	1.348 (10)
C13—C14	1.578 (12)	C89—N83	1.512 (10)
C13—C15	1.527 (10)	C89—C90	1.559 (12)
C13—H42	1.14 (4)	C89—C91	1.583 (10)
C14—C13	1.578 (12)	C89—H118	1.14 (3)
C14—C16	1.555 (10)	C90—C89	1.559 (12)
C14—H43	1.14 (6)	C90—C92	1.530 (8)
C14—H44	1.08 (6)	C90—H119	1.14 (4)
C15—N6	1.496 (9)	C90—H120	1.14 (6)
C15—C13	1.527 (10)	C91—N82	1.492 (9)
C15—H45	1.14 (6)	C91—C89	1.583 (10)
C15—H46	1.14 (4)	C91—H121	1.14 (6)
C16—N6	1.494 (8)	C91—H122	1.14 (5)
C16—C14	1.555 (10)	C92—N82	1.463 (8)
C16—H47	1.14 (5)	C92—C90	1.530 (8)
C16—H48	1.15 (5)	C92—H123	1.14 (5)
C17—N6	1.463 (11)	C92—H124	1.14 (5)
C17—C20	1.407 (5)	C93—N82	1.468 (11)
C17—C21	1.428 (8)	C93—C96	1.410 (5)
C18—N7	1.52 (2)	C93—C97	1.397 (8)
C18—H49	1.14 (7)	C94—N83	1.479 (18)
C18—H50	1.14 (8)	C94—H125	1.14 (8)
C18—H51	1.14 (7)	C94—H126	1.14 (7)
C19—N7	1.38 (2)	C94—H127	1.14 (6)
C19—H52	1.14 (8)	C95—C41^iii^	1.16 (7)
C19—H53	1.14 (6)	C95—H76^iii^	0.89 (6)
C19—H54	1.14 (6)	C95—N83	1.465 (18)
C20—N8	1.467 (10)	C95—H128	1.14 (9)
C20—C17	1.407 (5)	C95—H129	1.14 (6)
C20—C22	1.407 (8)	C95—H130	1.14 (6)
C21—C17	1.428 (8)	C96—N84	1.430 (9)
C21—C23	1.420 (9)	C96—C93	1.410 (5)
C21—H55	1.14 (3)	C96—C98	1.408 (8)
C22—C20	1.407 (8)	C97—C93	1.397 (8)
C22—C24	1.398 (10)	C97—C99	1.402 (8)
C22—H56	1.14 (3)	C97—H131	1.14 (3)
C23—O3	1.395 (8)	C98—C96	1.408 (8)
C23—C21	1.420 (9)	C98—C100	1.397 (8)
C23—C24	1.413 (9)	C98—H132	1.14 (3)
C24—N9	1.482 (13)	C99—O79	1.405 (8)
C24—C22	1.398 (10)	C99—C97	1.402 (8)
C24—C23	1.413 (9)	C99—C100	1.401 (9)
C25—O4	1.263 (10)	C100—N85	1.426 (13)
C25—N8	1.413 (8)	C100—C98	1.397 (8)
C25—C30	1.491 (7)	C100—C99	1.401 (9)
C26—N9	1.425 (11)	C101—O80	1.228 (10)
C26—N10	1.384 (11)	C101—N84	1.331 (8)
C26—N12	1.347 (6)	C101—C106	1.473 (7)
C27—O5	1.424 (7)	C102—N85	1.401 (11)
C27—C29	1.529 (4)	C102—N86	1.380 (11)
C27—C32	1.524 (6)	C102—N88	1.346 (6)
C27—C33	1.524 (6)	C103—O81	1.439 (8)
C28—O3	1.423 (14)	C103—C105	1.548 (5)
C28—H59	1.14 (9)	C103—C108	1.528 (7)
C28—H60	1.14 (8)	C103—C109	1.525 (7)
C28—H61	1.14 (4)	C104—O79	1.481 (14)
C29—C27	1.529 (4)	C104—H135	1.14 (6)
C29—C31	1.402 (5)	C104—H136	1.14 (8)
C29—C35	1.395 (7)	C104—H137	1.14 (10)
C30—C25	1.491 (7)	C105—C103	1.548 (5)
C30—C41	1.41 (3)	C105—C107	1.411 (5)
C30—H62	1.14 (3)	C105—C111	1.436 (17)
C31—N11	1.397 (13)	C106—C101	1.473 (7)
C31—C29	1.402 (5)	C106—C117	1.26 (2)
C31—C36	1.416 (8)	C106—H138	1.14 (4)
C32—C27	1.524 (6)	C107—N87	1.458 (13)
C32—H63	1.14 (5)	C107—C105	1.411 (5)
C32—H64	1.14 (7)	C107—C112	1.401 (8)
C32—H65	1.14 (7)	C108—C103	1.528 (7)
C33—C27	1.524 (6)	C108—H139^iv^	1.13 (6)
C33—H66	1.14 (3)	C108—H140^iv^	1.14 (6)
C33—H67	1.14 (8)	C108—H141^iv^	1.15 (7)
C33—H68	1.14 (6)	C109—C103	1.525 (7)
C34—N10	1.354 (13)	C109—H142^iv^	1.14 (6)
C34—N11	1.390 (9)	C109—H143^iv^	1.14 (6)
C34—C38	1.391 (10)	C109—H144^iv^	1.14 (6)
C35—C29	1.395 (7)	C110—N86	1.369 (9)
C35—C37	1.399 (9)	C110—N87	1.415 (9)
C35—H69	1.14 (2)	C110—C114	1.400 (10)
C36—C31	1.416 (8)	C111—C105	1.436 (17)
C36—C39	1.408 (10)	C111—C113	1.391 (14)
C36—H71	1.14 (2)	C111—H145	1.14 (3)
C37—F2	1.392 (17)	C112—C107	1.401 (8)
C37—C35	1.399 (9)	C112—C115	1.384 (8)
C37—C39	1.406 (8)	C112—H147	1.14 (3)
C38—C34	1.391 (10)	C113—F78	1.370 (18)
C38—C40	1.387 (9)	C113—C111	1.391 (14)
C38—H73	1.14 (3)	C113—C115	1.387 (8)
C39—Cl1	1.793 (11)	C114—C110	1.400 (10)
C39—C36	1.408 (10)	C114—C116	1.391 (9)
C39—C37	1.406 (8)	C114—H149	1.13 (3)
C40—N12	1.334 (10)	C115—Cl77	1.791 (11)
C40—C38	1.387 (9)	C115—C112	1.384 (8)
C40—H74	1.14 (2)	C115—C113	1.387 (8)
C41—C30	1.41 (3)	C116—N88	1.348 (10)
C41—H75	1.14 (6)	C116—C114	1.391 (9)
C41—H76	1.14 (5)	C116—H150	1.13 (3)
C41—C95^i^	1.16 (7)	C117—C106	1.26 (2)
C41—H130^i^	0.53 (10)	C117—H151	1.14 (8)
H42—C13	1.14 (4)	C117—H152	1.14 (5)
H43—C14	1.14 (6)	H118—C89	1.14 (3)
H44—C14	1.08 (6)	H119—C90	1.14 (4)
H45—C15	1.14 (6)	H120—C90	1.14 (6)
H46—C15	1.14 (4)	H121—C91	1.14 (6)
H47—C16	1.14 (5)	H122—C91	1.14 (5)
H48—C16	1.15 (5)	H123—C92	1.14 (5)
H49—C18	1.14 (7)	H124—C92	1.14 (5)
H50—C18	1.14 (8)	H125—C94	1.14 (8)
H51—C18	1.14 (7)	H126—C94	1.14 (7)
H52—C19	1.14 (8)	H127—C94	1.14 (6)
H53—C19	1.14 (6)	H128—C95	1.14 (9)
H54—C19	1.14 (6)	H129—C95	1.14 (6)
H55—C21	1.14 (3)	H130—C41^iii^	0.53 (10)
H56—C22	1.14 (3)	H130—C95	1.14 (6)
H57—N8	1.11 (3)	H131—C97	1.14 (3)
H58—N9	1.11 (3)	H132—C98	1.14 (3)
H59—C28	1.14 (9)	H133—N84	1.11 (3)
H60—C28	1.14 (8)	H134—N85	1.11 (3)
H61—C28	1.14 (4)	H135—C104	1.14 (6)
H62—C30	1.14 (3)	H136—C104	1.14 (8)
H63—C32	1.14 (5)	H137—H73^v^	0.523
H64—C32	1.14 (7)	H137—C104	1.14 (10)
H65—C32	1.14 (7)	H138—C106	1.14 (4)
H66—C33	1.14 (3)	H139—C108^iv^	1.13 (6)
H67—C33	1.14 (8)	H140—C108^iv^	1.14 (6)
H68—C33	1.14 (6)	H141—C108^iv^	1.15 (7)
H69—C35	1.14 (2)	H142—C109^iv^	1.14 (6)
H70—N11	1.11 (3)	H143—C109^iv^	1.14 (6)
H71—C36	1.14 (2)	H144—C109^iv^	1.14 (6)
H72—O5	1.11 (5)	H145—C111	1.14 (3)
H73—C38	1.14 (3)	H146—N87	1.11 (3)
H73—H137^ii^	0.523	H147—C112	1.14 (3)
H74—C40	1.14 (2)	H148—O81	1.09 (5)
H75—C41	1.14 (6)	H149—C114	1.13 (3)
H76—C41	1.14 (5)	H150—C116	1.13 (3)
H76—N83^i^	1.30 (4)	H151—C117	1.14 (8)
H76—C95^i^	0.89 (6)	H152—C117	1.14 (5)
			
C23—O3—C28	118.9 (7)	C91—N82—C92	109.1 (8)
C27—O5—H72	110 (3)	C91—N82—C93	129.7 (12)
C15—N6—C16	110.7 (8)	C92—N82—C93	120.7 (11)
C15—N6—C17	117.2 (11)	H76^iii^—N83—C89	84.9 (19)
C16—N6—C17	127.7 (13)	H76^iii^—N83—C94	140 (4)
C13—N7—C18	113.0 (10)	C89—N83—C94	108.4 (10)
C13—N7—C19	110.3 (10)	H76^iii^—N83—C95	37 (2)
C18—N7—C19	103.9 (15)	C89—N83—C95	115.8 (10)
C20—N8—C25	134.2 (10)	C94—N83—C95	107.7 (15)
C20—N8—H57	120 (2)	C96—N84—C101	122.8 (9)
C25—N8—H57	105.9 (18)	C96—N84—H133	120 (2)
C24—N9—C26	137.8 (14)	C101—N84—H133	117 (2)
C24—N9—H58	120 (2)	C100—N85—C102	129.7 (14)
C26—N9—H58	103 (2)	C100—N85—H134	120 (3)
C26—N10—C34	115.4 (5)	C102—N85—H134	110 (3)
C31—N11—C34	125.5 (14)	C102—N86—C110	116.1 (4)
C31—N11—H70	120 (2)	C107—N87—C110	128.0 (15)
C34—N11—H70	115 (2)	C107—N87—H146	120 (2)
C26—N12—C40	116.0 (5)	C110—N87—H146	112 (3)
N7—C13—C15	110.5 (13)	C102—N88—C116	115.7 (4)
N7—C13—H42	107 (2)	N83—C89—C90	109.5 (11)
C15—C13—H42	107 (3)	N83—C89—H118	108 (2)
C16—C14—H43	112 (5)	C90—C89—H118	108 (4)
C16—C14—H44	104 (4)	C89—C90—C92	102.3 (9)
H43—C14—H44	120 (2)	C89—C90—H119	111 (4)
N6—C15—C13	103.5 (7)	C92—C90—H119	111 (3)
N6—C15—H45	109 (3)	C89—C90—H120	109 (3)
C13—C15—H45	112 (3)	C92—C90—H120	113 (3)
N6—C15—H46	111 (3)	H119—C90—H120	111 (3)
C13—C15—H46	110 (4)	N82—C91—H121	110 (4)
H45—C15—H46	110 (3)	N82—C91—H122	111 (3)
N6—C16—C14	104.1 (4)	H121—C91—H122	110 (3)
N6—C16—H47	111 (3)	N82—C92—C90	103.2 (4)
C14—C16—H47	110 (4)	N82—C92—H123	110 (3)
N6—C16—H48	109 (3)	C90—C92—H123	111 (3)
C14—C16—H48	112 (4)	N82—C92—H124	109 (4)
H47—C16—H48	110 (2)	C90—C92—H124	113 (3)
N6—C17—C20	115.6 (6)	H123—C92—H124	111 (2)
N6—C17—C21	124.6 (6)	N82—C93—C96	120.5 (7)
C20—C17—C21	119.8 (5)	N82—C93—C97	120.9 (7)
N7—C18—H49	109 (6)	C96—C93—C97	118.3 (5)
N7—C18—H50	109 (4)	N83—C94—H125	109 (5)
H49—C18—H50	109 (3)	N83—C94—H126	110 (5)
N7—C18—H51	110 (2)	H125—C94—H126	110 (4)
H49—C18—H51	110 (6)	N83—C94—H127	109 (3)
H50—C18—H51	110 (7)	H125—C94—H127	109 (6)
N7—C19—H52	110 (6)	H126—C94—H127	110 (5)
N7—C19—H53	110 (4)	C41^iii^—C95—H76^iii^	66 (5)
H52—C19—H53	109 (4)	C41^iii^—C95—N83	120 (4)
N7—C19—H54	110 (3)	H76^iii^—C95—N83	61 (3)
H52—C19—H54	109 (5)	C41^iii^—C95—H128	83 (8)
H53—C19—H54	109 (6)	H76^iii^—C95—H128	129 (8)
N8—C20—C17	118.1 (7)	N83—C95—H128	109 (4)
N8—C20—C22	123.4 (9)	C41^iii^—C95—H129	122 (7)
C17—C20—C22	118.2 (5)	H76^iii^—C95—H129	121 (9)
C17—C21—C23	119.5 (10)	N83—C95—H129	110 (5)
C17—C21—H55	119.8 (15)	H128—C95—H129	109 (4)
C23—C21—H55	120.8 (16)	C41^iii^—C95—H130	27 (5)
C20—C22—C24	122.7 (7)	H76^iii^—C95—H130	49 (3)
C20—C22—H56	120.0 (18)	N83—C95—H130	110 (3)
C24—C22—H56	117.3 (17)	H128—C95—H130	109 (6)
O3—C23—C21	125.4 (6)	H129—C95—H130	110 (6)
O3—C23—C24	114.4 (6)	N84—C96—C93	120.5 (8)
C21—C23—C24	120.2 (5)	N84—C96—C98	120.1 (9)
N9—C24—C22	123.7 (10)	C93—C96—C98	119.3 (5)
N9—C24—C23	118.1 (8)	C93—C97—C99	121.9 (6)
C22—C24—C23	118.1 (6)	C93—C97—H131	120.0 (18)
O4—C25—N8	123.8 (6)	C99—C97—H131	118.1 (18)
O4—C25—C30	120.4 (7)	C96—C98—C100	122.1 (6)
N8—C25—C30	114.5 (3)	C96—C98—H132	120.0 (18)
N9—C26—N10	118.4 (14)	C100—C98—H132	117.9 (18)
N9—C26—N12	111.0 (12)	O79—C99—C97	124.6 (6)
N10—C26—N12	125.6 (4)	O79—C99—C100	115.5 (5)
O5—C27—C29	102.2 (8)	C97—C99—C100	119.9 (4)
O5—C27—C32	110.5 (9)	N85—C100—C98	122.6 (9)
C29—C27—C32	110.9 (7)	N85—C100—C99	119.1 (8)
O5—C27—C33	109.2 (9)	C98—C100—C99	118.2 (5)
C29—C27—C33	112.9 (7)	O80—C101—N84	122.2 (6)
C32—C27—C33	110.7 (6)	O80—C101—C106	123.7 (7)
O3—C28—H59	109 (6)	N84—C101—C106	113.6 (4)
O3—C28—H60	110 (6)	N85—C102—N86	119.9 (12)
H59—C28—H60	109 (4)	N85—C102—N88	112.7 (12)
O3—C28—H61	110 (3)	N86—C102—N88	125.8 (4)
H59—C28—H61	109 (6)	O81—C103—C105	111.9 (8)
H60—C28—H61	110 (6)	O81—C103—C108	104.8 (9)
C27—C29—C31	120.9 (7)	C105—C103—C108	112.2 (7)
C27—C29—C35	119.2 (7)	O81—C103—C109	104.7 (9)
C31—C29—C35	119.7 (5)	C105—C103—C109	112.7 (7)
C25—C30—C41	121.7 (8)	C108—C103—C109	110.1 (6)
C25—C30—H62	120 (2)	O79—C104—H135	109 (5)
C41—C30—H62	118 (2)	O79—C104—H136	109 (4)
N11—C31—C29	119.3 (6)	H135—C104—H136	110 (7)
N11—C31—C36	120.0 (9)	O79—C104—H137	109 (7)
C29—C31—C36	120.1 (5)	H135—C104—H137	110 (5)
C27—C32—H63	110 (3)	H136—C104—H137	109 (5)
C27—C32—H64	110 (4)	C103—C105—C107	125.1 (7)
H63—C32—H64	109 (5)	C103—C105—C111	116.2 (10)
C27—C32—H65	110 (4)	C107—C105—C111	117.0 (8)
H63—C32—H65	109 (6)	C101—C106—C117	122.2 (6)
H64—C32—H65	109 (3)	C101—C106—H138	120 (4)
C27—C33—H66	109 (3)	C117—C106—H138	118 (4)
C27—C33—H67	109 (3)	N87—C107—C105	121.7 (6)
H66—C33—H67	110 (5)	N87—C107—C112	117.6 (8)
C27—C33—H68	109 (4)	C105—C107—C112	120.4 (5)
H66—C33—H68	109 (5)	C103—C108—H139^iv^	110 (3)
H67—C33—H68	110 (3)	C103—C108—H140^iv^	110 (3)
N10—C34—N11	122.9 (15)	H139^iv^—C108—H140^iv^	110 (5)
N10—C34—C38	122.6 (7)	C103—C108—H141^iv^	109 (4)
N11—C34—C38	114.1 (11)	H139^iv^—C108—H141^iv^	109 (4)
C29—C35—C37	118.9 (8)	H140^iv^—C108—H141^iv^	109 (4)
C29—C35—H69	119.8 (16)	C103—C109—H142^iv^	109 (3)
C37—C35—H69	121.3 (16)	C103—C109—H143^iv^	110 (4)
C31—C36—C39	119.1 (9)	H142^iv^—C109—H143^iv^	109 (4)
C31—C36—H71	120.0 (15)	C103—C109—H144^iv^	110 (3)
C39—C36—H71	120.9 (14)	H142^iv^—C109—H144^iv^	109 (5)
F2—C37—C35	118.8 (10)	H143^iv^—C109—H144^iv^	109 (4)
F2—C37—C39	119.3 (7)	N86—C110—N87	119.5 (12)
C35—C37—C39	121.7 (5)	N86—C110—C114	121.9 (5)
C34—C38—C40	116.4 (3)	N87—C110—C114	117.9 (14)
C34—C38—H73	120 (2)	C105—C111—C113	120.1 (11)
C40—C38—H73	123 (2)	C105—C111—H145	120.2 (16)
Cl1—C39—C36	120.1 (6)	C113—C111—H145	119.8 (17)
Cl1—C39—C37	121.1 (6)	C107—C112—C115	121.2 (5)
C36—C39—C37	118.8 (5)	C107—C112—H147	120.1 (13)
N12—C40—C38	123.8 (6)	C115—C112—H147	118.7 (13)
N12—C40—H74	120 (3)	F78—C113—C111	119.7 (9)
C38—C40—H74	116 (3)	F78—C113—C115	119.1 (6)
C30—C41—H75	120 (4)	C111—C113—C115	120.2 (8)
C30—C41—H76	120 (4)	C110—C114—C116	116.3 (3)
H75—C41—H76	120 (4)	C110—C114—H149	120 (3)
C30—C41—C95^i^	164 (4)	C116—C114—H149	123 (3)
H75—C41—C95^i^	75 (4)	Cl77—C115—C112	118.4 (8)
H76—C41—C95^i^	45 (4)	Cl77—C115—C113	122.0 (6)
C30—C41—H130^i^	100 (7)	C112—C115—C113	119.0 (6)
H75—C41—H130^i^	121 (10)	N88—C116—C114	124.2 (4)
H76—C41—H130^i^	46 (3)	N88—C116—H150	120 (3)
C95^i^—C41—H130^i^	74 (7)	C114—C116—H150	115 (3)
C38—H73—H137^ii^	107.8 (17)	C106—C117—H151	120 (3)
C41—H76—N83^i^	138 (5)	C106—C117—H152	120 (5)
C41—H76—C95^i^	68 (5)	H151—C117—H152	120 (5)
N83^i^—H76—C95^i^	82 (3)	C41^iii^—H130—C95	79 (5)
C99—O79—C104	120.3 (7)	H73^v^—H137—C104	141 (2)
C103—O81—H148	112 (3)		

Symmetry codes: (i) *x*, *y*, *z*+1; (ii) *x*, *y*−1, *z*+1; (iii) *x*, *y*, *z*−1; (iv) −*x*+1, *y*, −*z*+1; (v) *x*, *y*+1, *z*−1.

### (_I_VASP) Crystal data

**Table:**

C_29_H_35_ClFN_7_O_3_	*c* = 19.85680 Å
*M**_r_* = 584.09	β = 117.25°
Monoclinic, *C*2	*V* = 6047.72 Å^3^
*a* = 33.50300 Å	*Z* = 8
*b* = 10.22560 Å	*T* = 298 K

### (_I_VASP) Data collection

**Table:**

*h* = →	*l* = →
*k* = →	

### (_I_VASP) Fractional atomic coordinates and isotropic or equivalent isotropic displacement parameters (Å^2^)

**Table:**

	*x*	*y*	*z*	*B*_iso_*/*B*_eq_	
Cl1	0.59812	−0.58413	1.04564		
F2	0.67191	−0.45003	1.17408		
O3	0.48377	0.11957	0.91453		
O4	0.24456	−0.10020	0.64500		
O5	0.55683	−0.33363	1.30971		
N6	0.34915	0.24619	0.68556		
N7	0.25686	0.39103	0.53685		
N8	0.30706	0.02593	0.70343		
N9	0.44324	−0.08017	0.93775		
N10	0.47122	−0.21298	1.04107		
N11	0.50242	−0.34843	1.14852		
N12	0.39118	−0.17917	0.97070		
C13	0.29793	0.41198	0.60711		
C14	0.34032	0.40473	0.59514		
C15	0.30789	0.31617	0.67390		
C16	0.37561	0.33763	0.66573		
C17	0.37152	0.16903	0.75081		
C18	0.21686	0.43020	0.54248		
C19	0.25144	0.26033	0.50490		
C20	0.34924	0.06059	0.76289		
C21	0.41683	0.19026	0.80230		
C22	0.37171	−0.01886	0.82696		
C23	0.43926	0.10877	0.86452		
C24	0.41657	0.00336	0.87852		
C25	0.27296	−0.04877	0.70421		
C26	0.43381	−0.16071	0.98354		
C27	0.58156	−0.24716	1.28306		
C28	0.51074	0.20717	0.89716		
C29	0.58489	−0.31881	1.21824		
C30	0.27152	−0.05665	0.77750		
C31	0.54590	−0.36567	1.15499		
C32	0.55491	−0.12013	1.25828		
C33	0.62710	−0.21618	1.34960		
C34	0.46524	−0.29475	1.08812		
C35	0.62677	−0.34805	1.22221		
C36	0.55051	−0.44482	1.10127		
C37	0.63047	−0.42375	1.16747		
C38	0.42180	−0.32321	1.07900		
C39	0.59247	−0.47610	1.10733		
C40	0.38656	−0.26106	1.01980		
C41	0.24525	−0.14408	0.78792		
H42	0.29476	0.51106	0.62589		
H43	0.33326	0.34638	0.54452		
H44	0.35074	0.50212	0.58595		
H45	0.31333	0.36986	0.72572		
H46	0.27991	0.24813	0.66079		
H47	0.40112	0.28441	0.65612		
H48	0.39327	0.41025	0.71164		
H49	0.20774	0.36284	0.57728		
H50	0.18818	0.43304	0.48549		
H51	0.22121	0.52838	0.56744		
H52	0.24297	0.18481	0.53667		
H53	0.28182	0.22898	0.50168		
H54	0.22432	0.26225	0.44648		
H55	0.43468	0.27194	0.79344		
H56	0.35544	−0.10530	0.83494		
H57	0.30306	0.04551	0.65014		
H58	0.47590	−0.08894	0.94689		
H59	0.54465	0.19813	0.94426		
H60	0.49945	0.30937	0.89369		
H61	0.51099	0.17942	0.84370		
H62	0.29230	0.01186	0.82197		
H63	0.55111	−0.07949	1.30615		
H64	0.57357	−0.04974	1.24200		
H65	0.52153	−0.13315	1.21031		
H66	0.64678	−0.15019	1.33264		
H67	0.64725	−0.30453	1.37436		
H68	0.62151	−0.16672	1.39363		
H69	0.65777	−0.31237	1.26876		
H70	0.50014	−0.35770	1.19782		
H71	0.52052	−0.48723	1.05572		
H72	0.57605	−0.40921	1.33311		
H73	0.41675	−0.39392	1.11529		
H74	0.35208	−0.27899	1.00967		
H75	0.22587	−0.21258	0.74259		
H76	0.24209	−0.14828	0.84010		
Cl77	0.55656	0.75824	0.74883		
F78	0.55305	1.04286	0.75287		
O79	0.44002	0.44922	0.20978		
O80	0.33849	0.04332	0.32855		
O81	0.42718	1.08565	0.42668		
N82	0.32255	0.12378	0.08011		
N83	0.22651	0.04533	−0.06503		
N84	0.31064	0.10325	0.20321		
N85	0.40961	0.48806	0.31334		
N86	0.42039	0.65827	0.39359		
N87	0.43505	0.83465	0.47409		
N88	0.37256	0.47958	0.38964		
C89	0.27285	0.07837	−0.04820		
C90	0.30577	−0.03757	−0.01238		
C91	0.29549	0.19141	0.00836		
C92	0.34424	0.01576	0.06048		
C93	0.34945	0.20495	0.14327		
C94	0.21833	0.03461	0.00058		
C95	0.19350	0.13005	−0.12233		
C96	0.34142	0.19993	0.20704		
C97	0.38290	0.28846	0.14539		
C98	0.36108	0.29045	0.26621		
C99	0.40504	0.37147	0.20649		
C100	0.39139	0.38298	0.26411		
C101	0.31233	0.02523	0.26036		
C102	0.39973	0.54231	0.36764		
C103	0.42947	1.11606	0.49959		
C104	0.48330	0.40042	0.26343		
C105	0.46308	1.02295	0.55845		
C106	0.28043	−0.08639	0.23358		
C107	0.46197	0.88571	0.54653		
C108	0.44342	1.25966	0.51646		
C109	0.38361	1.09639	0.49826		
C110	0.41756	0.71057	0.45322		
C111	0.49476	1.07227	0.62799		
C112	0.49115	0.80379	0.60508		
C113	0.52343	0.99041	0.68503		
C114	0.39370	0.64537	0.48653		
C115	0.52154	0.85585	0.67458		
C116	0.36994	0.53492	0.44917		
C117	0.28665	−0.19255	0.27662		
H118	0.27110	0.10482	−0.10314		
H119	0.28806	−0.11692	0.00022		
H120	0.31819	−0.07668	−0.05072		
H121	0.27180	0.25913	0.01425		
H122	0.31669	0.24965	−0.01024		
H123	0.35842	−0.05560	0.10698		
H124	0.37214	0.05112	0.04970		
H125	0.22127	0.12902	0.03036		
H126	0.18415	−0.00282	−0.01754		
H127	0.24198	−0.03487	0.04180		
H128	0.15958	0.09392	−0.13702		
H129	0.19531	0.23381	−0.10403		
H130	0.19781	0.12847	−0.17396		
H131	0.39247	0.28871	0.09974		
H132	0.35212	0.29403	0.31223		
H133	0.29431	0.06747	0.14871		
H134	0.42803	0.55061	0.29840		
H135	0.48711	0.40248	0.32163		
H136	0.50846	0.46489	0.26003		
H137	0.48795	0.29953	0.24894		
H138	0.25225	−0.08029	0.17660		
H139	0.47778	1.27479	0.52453		
H140	0.44124	1.29561	0.56696		
H141	0.42031	1.31793	0.46796		
H142	0.35855	1.16325	0.45728		
H143	0.37165	0.99595	0.48169		
H144	0.38641	1.11568	0.55474		
H145	0.49788	1.17687	0.63882		
H146	0.43381	0.89455	0.43169		
H147	0.49126	0.69904	0.59557		
H148	0.39525	1.08678	0.38790		
H149	0.39087	0.68730	0.53448		
H150	0.34669	0.48739	0.46602		
H151	0.26401	−0.27621	0.25675		
H152	0.31520	−0.19854	0.33299		

### (_I_VASP) Hydrogen-bond geometry (Å, º)

**Table:**

*D*—H···*A*	*D*—H	H···*A*	*D*···*A*	*D*—H···*A*
N9—H58···N10^i^	1.03	2.10	3.022	148
N11—H70···O5	1.02	2.18	2.872	124
N11—H70···Cl77^ii^	1.02	2.82	3.597	133
N85—H134···Cl77^iii^	1.02	2.47	3.424	155
N87—H146···O81	1.03	1.96	2.705	127
O81—H148···O80^iv^	0.99	1.77	2.740	164
C38—H73···O79^v^	1.09	2.32	3.329	154
C90—H120···N12^vi^	1.10	2.51	3.358	133
C104—H136···O5^vii^	1.10	2.53	3.498	147
C108—H141···N88^iv^	1.10	2.33	3.400	165

Symmetry codes: (i) −*x*+1, *y*, −*z*+2; (ii) −*x*+1, *y*−1, −*z*+2; (iii) −*x*+1, *y*, −*z*+1; (iv) *x*, *y*+1, *z*; (v) *x*, *y*−1, *z*+1; (vi) *x*, *y*, *z*−1; (vii) *x*, *y*+1, *z*−1.
